# Effect of deprivation and ethnicity on primary macula-on retinal detachment repair success rate and clinical outcomes: A study of 568 patients

**DOI:** 10.1371/journal.pone.0259714

**Published:** 2021-11-09

**Authors:** George Moussa, Dimitrios Kalogeropoulos, Soon Wai Ch’ng, Kim Son Lett, Arijit Mitra, Ajai K. Tyagi, Ash Sharma, Walter Andreatta

**Affiliations:** 1 Birmingham Midlands Eye Centre, Sandwell and West Birmingham Hospitals NHS Trust, Birmingham, United Kingdom; 2 Birmingham and Midland Eye Centre and Academic Unit of Ophthalmology, University of Birmingham, Birmingham, United Kingdom; 3 Department of Ophthalmology, Faculty of Medicine, School of Health Sciences, University of Ioannina, Ioannina, Greece; 4 Kantonsspital Winterthur, Winterthur, Switzerland; 5 University of Zurich, Zurich, Switzerland; Medical Research Foundation (Sankara Nethralaya), INDIA

## Abstract

**Purpose:**

Socio-economic deprivation and ethnic variation have been frequently linked to poorer health outcomes. We collected a large series of primary macula-on rhegmatogenous retinal detachment (RRD) cases and analysed the effect of socio-economic deprivation and ethnicity on both six-month retinal re-detachment rate and visual outcomes.

**Materials and methods:**

Retrospective consecutive case series of 568 patients attending Birmingham and Midlands Eye Centre from January 2017–2020. Multiple Indices of Deprivation (IMD) deciles were used for deprivation status and split to two groups: IMD-A (Decile 1–5) and IMD-B (Decile 6–10). The two largest subgroups of ethnicities were compared, White and South Asians (SA).

**Results:**

We report an overall retinal re-detachment rate of 8.5%. IMD-A re-detached significantly more than IMD-B (11.2% vs 6.0% respectively, p = 0.034). No statistical significance was found between White and SA re-detachment rate (9.1% and 5.6% respectively, p = 0.604). SA median age significantly lower at 49 years (IQR: 37–61) compared to White patients at 57 years (IQR: 50–65) (p = <0.001). IMD-A median age of 55 years (IQR: 46–64) was significantly lower to IMD-B median age of 58 years (IQR: 51–65) (p = 0.011). No differences in final visual outcomes were detected across all groups.

**Conclusion:**

We demonstrated an increased retinal re-detachment rate in our more deprived patients according to IMD and a younger cohort of SA compared to White ethnicity. Further prospective studies are required to demonstrate the link between socio-economic deprivation and surgical success.

## Introduction

Rhegmatogenous retinal detachment (RRD) is one of the most common surgical ophthalmic emergencies in the United Kingdom [1]. Saidkasimova et al. reported an higher incidence of RRD in affluent areas, possibly due to the higher rate of cataract surgeries performed. On the other hand, Mitry et al. found that more deprived patients were more likely to present with macula-off RRD compared to the most affluent ones [2, 3].

Low socio-economic status is associated with an increased incidence of blindness and visual impairment due to a variety of ocular pathologies other than RRD [4, 5] In addition, three out of six studies reported in a systematic review by Lane et al. found significantly better outcomes in cataract surgery in more affluent individuals [6].

To the best of our knowledge, no previous study has yet determined the effect of social deprivation on the success rate and final visual acuity (VA) following RRD repair. We therefore proceeded to investigate this association in our study [9]. In addition, we explored effect of ethnicity on surgical outcomes in RRD. The West Midlands region has the second highest ethnic variability in the United Kingdom (UK) according to the 2011 census and represents the ideal location for such a study [7].

## Materials and methods

This is a retrospective consecutive case series of 568 patients operated by vitreoretinal fellows and consultants at the Birmingham and Midlands Eye Centre (BMEC), UK from January 2017 to 2020. The research adhered to the tenets of the Declaration of Helsinki and all patient data extracted were anonymised for analysis.

Our primary outcome measures were the retinal re-attachment rate and visual outcomes following RRD repair. Only cases presenting with primary macula-on RRD without proliferative vitreoretinopathy (PVR) were included to minimise confounding variables in assessing visual outcomes (i.e. macula-off status) and to adjust for case complexity [8]. These were inclusive of all cases repaired by pars plana vitrectomy (PPV), scleral buckle (SB) or pneumatic retinopexy.

All data for primary macula-on RRD were extracted from electronic patient records (EPR, Medisoft Ophthalmology, Medisoft Limited, Leeds, UK). All secondary RRD surgery were excluded. Retinal re-detachment rates were based on repeat RRD surgery in the same eye within six-months. Six-months was chosen to include late re-detachments in line with other published work [9, 10] and allow sufficient time following complete reabsorption of the gas intraocular tamponade. All PPV were performed with transconjunctival 23-gauges with fluid air exchange (FAX) and vitreous-base trim performed. Retinopexy of retinal breaks was achieved with either external cryotherapy, laser retinopexy or a combination of these. All SB procedures were performed through standard indirect ophthalmoscope viewing system, cryotherapy and silicone tires. No patients underwent combined SB and PPV in our cohort.

### Deprivation ranking

Deprivation ranking in the study was based on the criteria available through gov.uk. [11]. The English Government has developed the English Indices of Deprivation 2019 (IoD) which are readily available data in the UK. The IoD are a unique measure of relative deprivation across England. These are based on seven different domains of deprivation which are combined to produce an overall weighted relative measure of deprivation, the Index of Multiple Deprivation (IMD): (weighting in brackets %) [11]

- Income Deprivation (22.5%)
- Employment Deprivation (22.5%)
- Education, Skills and Training Deprivation (13.5%)
- Health Deprivation and Disability (13.5%)
- Crime (9.3%)
- Barriers to Housing and Services (9.3%)
- Living Environment Deprivation (9.3%)

The IMD is given a rank against every postcode area in England. Following calculation of these ranks, a decile is calculated, one indicating highest level of deprivation and ten being the most affluent. The full postcode of patients was used to extract the IMD decile and correlate with our clinical outcomes of VA and re-detachment rate. To increase statistical power, IMD Decile was dichotomised for analysis: i) More deprived: Deciles 1–5 (IMD-A) and ii) Less deprived: Deciles 6–10 (IMD-B).

The following data were collated: age of patient, gender, ethnicity, preoperative and postoperative VA, surgery type, tamponade used (if applicable) and IMD decile rank. Ethnicity was self-reported by patients upon registration to our hospital system.

### Statistical analysis

Statistical significance was defined as p<0.05. Prior to analysis, normality of continuous variables was assessed using the Shapiro-Wilk test, and found not to be normally distributed. Hence, data are primarily reported as medians and interquartile ranges (IQRs) throughout the statistical analysis. Mann Whitney U was used to compare two independent groups respectively (age, and VA). Wilcoxon signed rank test was used for two-paired VA data. Fisher exact test and Chi-Squared test were used for nominal variables. Bonferroni correction was applied for multiple statistical analysis. Best corrected VA was used and records in Snellen were converted to LogMAR. Low VA, corresponding to count fingers (CF), hand movements (HM), perception of light (PL) and no PL (NPL) were substituted with 2.10, 2.40, 2.70 and 3.00 LogMAR, respectively, in keeping with previous publications from the national ophthalmology database group [12, 13]. All statistical analysis was performed using IBM SPSS Statistics for Windows, Version 25.0 (IBM Corp, Armonk NY).

### Ethical approval / consent to participation

This study was registered and approved by our local clinical effectiveness team (Clinical Effectiveness Department, Sandwell General Hospital: reference number: 1593). As this was a retrospective anonymized study, as per our local protocol from our Clinical Effectiveness Department, and as per national guidelines from the National Code of Clinical Research, and the Health Research Authority (HRA), this study has ethical approval exemption and no patient consent was required for participation [14, 15]. All procedures were completed prior to the design of this study. Patients were diagnosed and treated according to local guidelines and agreements and written consent from patients was acquired prior to all procedures as clinically indicated. This study does not report on the use of new or experimental protocols.

## Results

From the total of 568 cases, PPV, SB and pneumatic retinopexy were performed in 484 (85.2%), 80 (14.1%) and 4 (0.7%) cases respectively. The overall average success rate for primary RRD surgery was 91.5%. We had 353 (62.1%) males in our cohort. 384 (87.2%) individuals were White, 54 (9.5%) South Asian (SA), 13 (2.3%) Black and 14 (2.5%) other minor ethnic background.

Ethnicity data was missing in 103 cases and small numbers were present for Black and other minor ethnicities (n = 13 and n = 14 respectively). Therefore, only a comparison of clinical characteristics between SA (Pakistani, Indian and Bangladeshi) and White patients was performed across 437 patients.

### Effect of Indices of Multiple Deprivation decile

A summary of clinical characteristics according to deprivation status is found in Table 1. We found a significant difference in age between IMD-A and IMD-B (p = 0.011), with the IMD-A group being the younger group. We found a correlation between age and IMD decile in our cohort (R = 0.124, p = 0.003, Spearman’s Rank Correlation Co-efficient). As the cohort of patients are younger in IMD-A, they underwent a significantly higher proportion of SB compared to IMD-B group (p = 0.002). Within the PPV subgroup, a significantly longer acting gas tamponade and oil were used in IMD-A group compared to the IMD-B group (p = 0.012).

**Table 1:** Deprivation status and clinical characteristics of macula-on RRD repair.

	Total	More Deprived	Less Deprived	p-Value
		IMD-A	IMD-B	
Total	568	269	299	**-**
**Age (years)**	57.0 (49.0–65.0)	55.0 (46.0–64.0)	58.0 (51.0–65.0)	**0.011**
**Gender (% Male)**	353 (62.1%)	163 (60.6%)	190 (63.5%)	0.489
**Ocular Comorbidity**	117 (20.6%)	59 (21.9%)	58 (19.4%)	0.469
**(%Yes)**				
**Surgery Type**				
PPV	484 (85.2%)	218 (81.0%)	266 (89.0%)	**0.002** ^†^
SB	80 (14.1%)	51 (19.0%)	29 (9.7%)	
Pneumatic	4 (0.7%)	0 (0.0%)	4 (1.3%)	-
**Tamponade (PPV only)** *				-
SF_6_	222 (45.9%)	89 (40.8%)	133 (50.0%)	**0.012**
C_2_F_6_	122 (25.2%)	53 (24.3%)	69 (25.9%)	
C_3_F_8_	103 (21.3%)	52 (23.9%)	51 (19.2%)	
Oil	31 (6.4%)	22 (10.1%)	9 (3.4%)	
Silicone oil	12 (2.5%)	9 (4.1%)	3 (1.1%)	1.000
Densiron	19 (3.9%)	13 (6.0%)	6 (2.3%)	

Data are reported as median (interquartile range). Mann-Whitney U test was used to compare Age between groups. Chi-Squared (>2 groups) and fisher-exact test (2 groups) were otherwise used to compare nominal groups.

^†^Pneumatic cases excluded due to low numbers.

*Data unavailable in 6 patients.

Statistical significance in bold.

**Fig 1:**
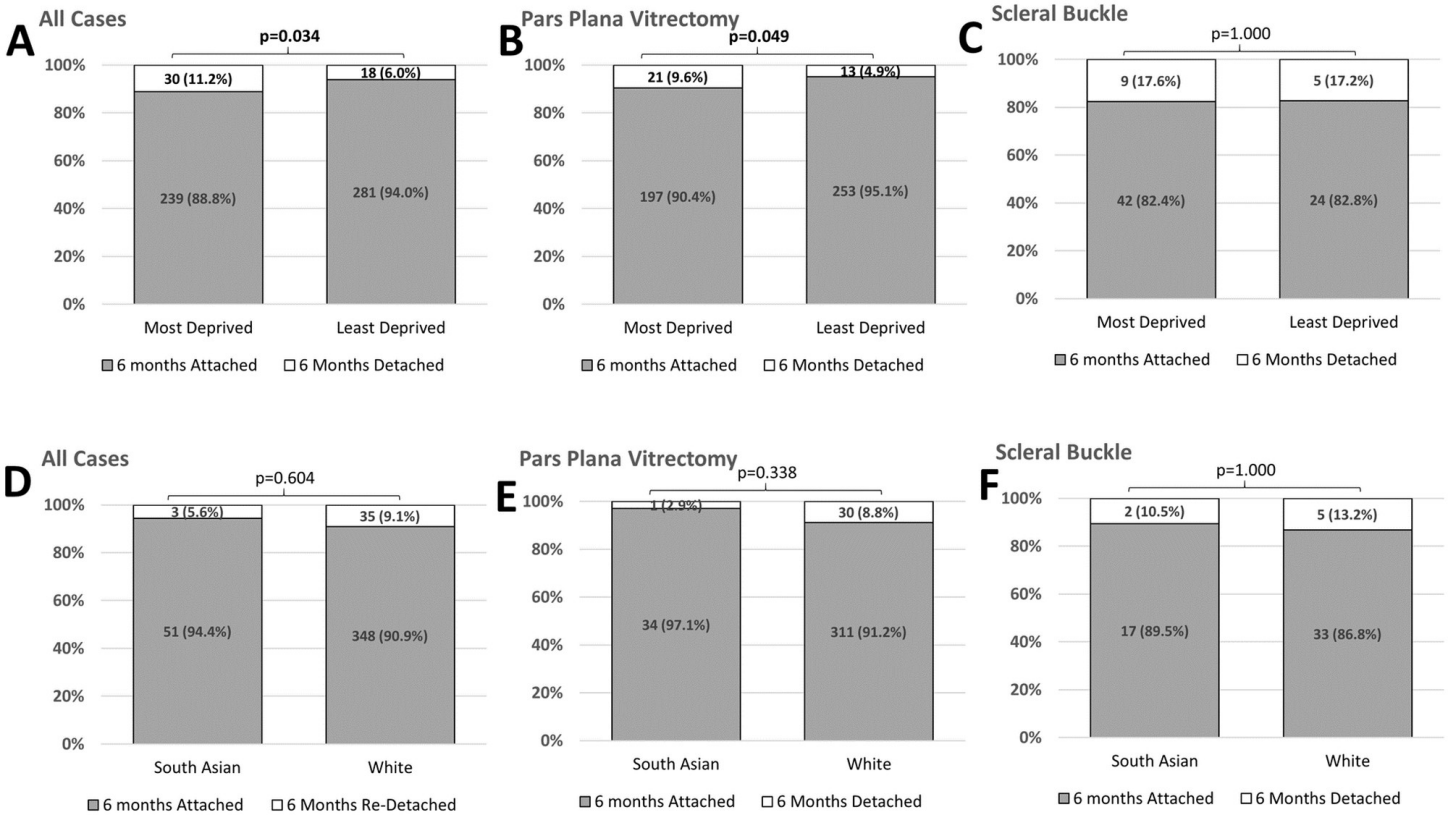
Re-detachment rate at six-months by ethnicity and deprivation status: Sub group analysis. Fisher exact test. Statistical significance in bold. A: Re-Detachment rate at six-months vs Deprivation Status for all cases. B: Re-Detachment rate at six-months vs Deprivation Status for Pars Plana Vitrectomy. C: Re-Detachment rate at six-months vs Deprivation Status for Scleral Buckle. D: Re-Detachment rate at six-months vs Ethnicity for all cases. E: Re-Detachment rate at six-months vs Ethnicity for Pars Plana Vitrectomy. F: Re-Detachment rate at six-months vs Ethnicity for Scleral Buckle. Significant found between deprivation status and six-month re-detachment rate for all cases and pars plana vitrectomy subgroup.

Across all operations, IMD-A had a higher proportion of six-month re-detachments (Fig 1A, p = 0.034). As our cohort had higher re-detachment rate in SB compared to PPV (Table 2, p = 0.004) and IMD-A group is significantly younger, subgroup analyses were performed on the PPV and SB group, separately. Fig 1B shows a significantly higher six-month re-detachment rate in IMD-A than IMD-B in the PPV sub-group (p = 0.049), but no difference in the SB (p = 1.000, Fig 1C).

**Table 2:** Comparing factors affecting six-month re-detachment rate.

Six-Month Re-detachment:	No n = 520 (91.5%)	Yes n = 48 (8.5%)	p-Value
**Age (years)** ^&^	57.0 (49.0–65.0)	55.5 (42.5–64.0)	0.106
<30	55 (10.6%)	9 (18.8%)	0.273
30–39	69 (13.3%)	10 (20.8%)	
40–49	65 (12.5%)	6 (12.5%)	
50–59	147 (28.3%)	9 (18.8%)	
60–69	115 (22.2%)	10 (20.8%)	
70–79	51 (9.8%)	4 (8.3%)	
80+	17 (3.3%)	0 (0.0%)	
**Gender (% Male)**	320 (61.5%)	33 (68.8%)	0.354
**Ocular Comorbidity (% Yes)**	106 (20.4%)	11 (22.9%)	0.709
**Surgery Type**			
PPV	450 (86.5%)	34 (70.8%)	**0.004** ^†^
SB	66 (12.7%)	14 (29.2%)	
Pneumatic	4 (0.8%)	0 (0.0%)	-
**Tamponade (PPV only)** *			
SF_6_	204 (45.3%)	18 (52.9%)	0.301
C_2_F_6_	118 (26.2%)	4 (11.8%)	
C_3_F_8_	94 (20.9%)	9 (26.5%)	
Oil	28 (6.2%)	3 (8.8%)	
Silicone oil	11 (2.4%)	1 (2.9%)	1.000
Densiron	17 (1.6%)	2 (5.9%)	
**Deprivation Status**			
More Deprived (IMD-A)	239 (46.0%)	30 (62.5%)	**0.034**
Less Deprived (IMD-B)	281 (54.0%)	18 (37.5%)	
**Ethnicity** **			
White	348 (83.1%)	35 (77.8%)	0.604^†^
South Asian	51 (12.2%)	3 (6.7%)	
Black	12 (2.9%)	1 (2.2%)	-
Other	8 (1.9%)	6 (13.3%)	-
**Visual Acuity**			
Pre-Op	0.18 (0.00 to 0.30)	0.18 (0.00 to 0.30)	0.844
Post-Op	0.18 (0.00 to 0.30)	0.48 (0.18 to 0.78)	**<0.001**
LogMAR gain	0.0 (-0.18 to 0.12)	-0.18 (-0.48 to 0.00)	**<0.001**

Data are reported as median (interquartile range). Mann Whitney U was used to compare continuous data (age, and visual acuity (VA)). Chi-Squared was otherwise used to compare nominal groups.

^†^pneumatic cases and ethnicities other than White and South Asian excluded due to low numbers.

*Data unavailable in six patients.

**Data unavailable in 103 of which 100 did not detach, and 3 detached.

^&^Data missing in one patient.

Although IMD-A had a higher re-detachment rate and a higher percentage of eyes requiring a longer acting tamponade, no correlation between primary success and tamponade used was found across both groups (p = 0.301).

### Differences among ethnicities

We found a statistically significant difference in the age of SA and White patients (p<0.001) and a significantly higher proportion of SB performed in the SA group (p<0.001) (Table 3). No difference was found in six-month re-detachment rate between SA and White patients across all cases (Fig 1D), PPV (Fig 1E) and SB subgroups (Fig 1F).

**Table 3:** Ethnicity (two largest subgroups) and clinical characteristics of macula-on RRD repair.

	Total	South Asian	White	p-Value
Total	437	54	383	**-**
**Age (years)**	57.0 (49.0–65.0)	49.0 (37.0–61.0)	57.0 (50.0–65.0)	**<0.001**
**Gender (% Male)**	265 (60.6%)	35 (64.8%)	230 (60.1%)	0.554
**Ocular Comorbidity (%Yes)**	86 (19.7%)	16 (29.6%)	70 (18.3%)	0.066
**Surgery Type**				
PPV	376 (86.0%)	35 (64.8%)	341 (89.0%)	**<0.001** ^†^
SB	57 (13.0%)	19 (35.2%)	38 (9.9%)	
Pneumatic	4 (0.9%)	0 (0.0%)	4 (1.0%)	-
**Tamponade (PPV only)** *				-
SF_6_	175 (46.5%)	8 (22.9%)	167 (49.0%)	**0.023**
C_2_F_6_	90 (23.9%)	11 (31.4%)	79 (23.2%)	
C_3_F_8_	85 (22.6%)	13 (37.1%)	72 (21.1%)	
Oil	20 (5.3%)	3 (8.6%)	17 (5.0%)	
SO	10 (2.7%)	2 (5.7%)	8 (2.3%)	1.000
Densiron	10 (2.7%)	1 (2.9%)	9 (2.6%)	

Data are reported as median (interquartile range). Mann-Whitney U test was used to compare Age between groups. Chi-Squared (>2 groups) and fisher-exact test (2 groups) were otherwise used to compare nominal groups.

^†^pneumatic cases excluded due to low numbers.

*Data unavailable in 6 patients.

Statistical significance in bold.

Within the PPV subgroup, significantly longer acting gas tamponade and oil were used in SA compared to the White subgroup (Table 3, p = 0.023).

Factors affecting six-month re-detachment rate are found in Table 2. SB had higher proportion of re-detachment than PPV (p = 0.004) and IMD-A was identified as statistically significant risk factor (p = 0.034).

We report our age by subcategories in Table 2. We found no difference in re-detachment rate by age. We analysed the effects of age and re-detachment rate by separating PPV and SB into different groups and found no difference in re-detachment rate (p = 0.718 and p = 0.882 respectively).

### Visual outcomes

**Fig 2:**
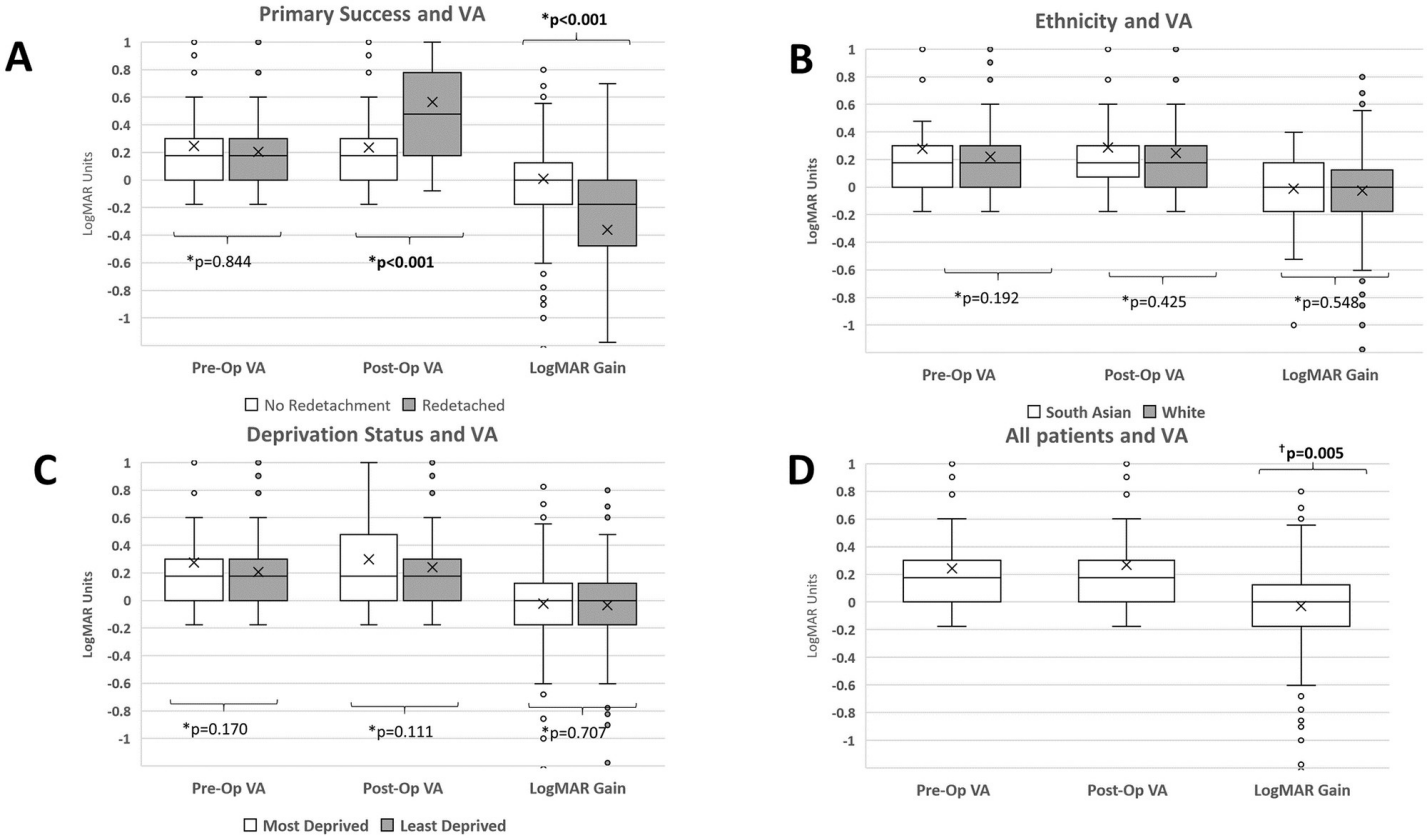
Box and Whisker plot. ‘X’ denotes mean. *Mann Whitney U. ^†^Wilcoxon-Signed Rank Test. Statistical significance in bold. VA: Visual Acuity. A: Primary Success and VA: worse VA for cases that re-detached at six-months vs. successful detachment repair. B: Ethnicity and VA. C: Deprivation status and VA. D: VA outcomes across all patients. Significantly worse Pre to Post-OP VA across all cases irrespective of detachment status.

Pre, post-operative VA and paired LogMAR gain in VA are summarised in Fig 2. No significant difference was found in deprivation status or ethnicity between pre-operative LogMAR, post-operative LogMAR and LogMAR gain. Patients that re-detached, had significantly worse post-operative LogMAR (p<0.001).

## Discussion

To our knowledge, this is the first study to assess the effect of socio-economic deprivation on surgical success and visual outcomes following RRD repair and we additionally reported on the effect of ethnicity on these outcomes. Although our surgical success of RRD repair was comparable to other large case series [16–19], we found that deprivation was associated with a lower success rate, as the re-detachment rate at six-months was higher in the most-deprived (21 cases, 9.6%) compared to the less deprived (13 cases, 4.9%) individuals. The more-deprived patients were more likely to have a SB, oil, or a long-acting tamponade. However, the choice of tamponade did not correlate to a higher re-detachment rate and therefore, was not found to contribute to the difference in re-detachment rate. Rather, the choice of longer acting gas or oil tamponade might be more likely attributed to the subjective opinion of the attending surgeon regarding the patient’s compliance with posturing rather than a link to the deprivation status. Having identified that SB have a higher re-detachment rate than PPV, we performed a subgroup analysis on the effect deprivation and re-detachment rate int he SB and PPV groups separately. In our PPV subgroup, more deprived patients have a significant higher re-detachment rate (p = 0.049). Although the more deprived group in our study population was younger, the was no overall significant difference on the effect of age for both PPV and SB re-detachment rate. Besides that, we identified no other baseline demographics or clinical characteristics that may explained the higher re-detachment rate in the more deprived group. The authors hypothesis this difference may be attributed to post-operative factors, such as posturing compliance. Additionally, patients in the deprived groups are more likely to have less social and/or occupational support to take sufficient sick leave to ensure an adequate post-operative recovery. Further prospective studies are required to further explore the differences measured in this study.

The effect of socio-economic status on morbidity and mortality has long been recognised within and outside the field of Ophthalmology. Although there was no report on outcomes, a Scottish cohort of 1,244 cases indicated that patients from more deprived areas affected by primary RRD seek medical help later than more affluent individuals with 50.8% of cases in the most affluent quintile presented with a detached or bisected macula compared with 65.0% in the more deprived quintile [20]. In New Zealand, Allbon et al. also reported on the delayed presentation of the more socially deprived group in their cohort for patients with RRD without any difference in visual outcomes or macula status in 94 patients [21]. As delayed presentation and macula status are crucial confounders in visual outcomes and primary success, our study only included primary macula-on RRD cases without PVR in order to minimise the confounding effects associated with a late presentation. The latter is known to increase case complexity which contributes to poorer surgical and visual outcomes [8]. Additionally, these criteria allowed more comparative assessments on the link between social deprivation and surgical outcomes by reducing the time factor of the presenting RRD, although we acknowledge some patients will have acute on chronic macula-on RD.

The impact of deprivation in the most common acquired causes of visual loss in adults, including cataract, diabetic eye disease, glaucoma, age-related macular degeneration, ocular trauma and even the financial burden of socio-economic deprivation in microbial keratitis [21] have also been explored [6]. Lane et al.’s systemic review found that low income and low educational attainment are linked with sight-threatening pathologies due to delays to specialist assessment and delivery of treatment [6]. Their study presents a cross-sectional model of the compound effects of deprivation. The significant effect of deprivation on human vision was also highlighted in the retrospective study by O’Colmain et al., who reported the outcomes of 430 children that did not pass Pre-School Vision Screening (PSVS) [22]. They found that children from adverse family backgrounds or socioeconomically deprived areas were more likely to miss appointments or to be lost to follow-up. These children were more than five times more likely to develop amblyopia and three times more likely to have lower or no binocular vision.

Similarly, on a global scale, cataract surgery rate is considerably higher in wealthy industrialized countries [23], as poverty and low socioeconomic status in developing countries was found to be obstructive to accessible healthcare [24, 25]. In the UK, where the provision of healthcare is free, there is evidence that social deprivation may affect access to cataract surgery [26, 27].

Similar to the report by Chandra et al. [28], we found that SA patients presented with RRD at a younger age compared to White patients. Gupta et al. also reported a younger age in SA and the increased likelihood to require longer acting tamponade than White patients [29]. In our cohort, although SA ethnicity was associated with a younger age [30], a higher SB rate, the usage of oil and long-acting tamponade, we did not encounter lower overall success rate relative to White patients. Both ethnicity and deprivation status did not affect the final LogMAR gain in VA.

### Study limitations and strengths

The limitations of our study include its retrospective nature and lack of case randomisation. Additionally, we used re-operation as a rate of failed detachment at six-months. Patients may have had their repeat surgery at another eye unit, although patients were excluded by postcode to minimize this probability. We used a full postcode to assess the IMD which is inherently an estimate of deprivation as some deprived households will reside in affluent areas, and affluent households in deprived areas. Ethnicity was self-reported, and this may introduce a source of bias, as some ethnicities may be less likely to volunteer such information, leading to possible underrepresentation of certain ethnicities. Additionally, mixed ethnicities are substantial proportion of patients in the UK and were not included in our analyses due to low statistical power of small subgroups. Despite this, our study has several strengths. The authors could not reference another study assessing the link between social deprivation and the surgical outcomes of RRD surgery. We risk stratified our cohort to allow for meaningful comparisons by including only primary macula-on RRDs although we acknowledge that there are variations in chronicity and therefore management even in this subgroup of RD repairs. The retrospective approach allowed us to collect a large number of cases which allowed adequate numbers to perform subgroup analysis.

## Conclusions

This study has shown there is a causal relationship between social deprivation and an increased risk for surgical failure in RRD repair. However, a prospective study is required to determine a possible causative effect. This study also highlights that health disparities may always persist, especially in specific geographic areas, but an early and increased recognition of its socio-economic impact may urge policy makers to provide more resources to the most vulnerable groups and improve their quality of life.

## Supporting information

S1 File Raw data used in our manuscript. (XLSX)
